# Association between abstraction level and time: Are future and past more abstract than the present?

**DOI:** 10.1177/17470218231217732

**Published:** 2023-12-27

**Authors:** Karin M Bausenhart, Rolf Ulrich, Barbara Kaup

**Affiliations:** University of Tübingen, Tübingen, Germany

**Keywords:** Representation format, construal level, temporal cognition, abstraction

## Abstract

Construal level theory suggests that objects or events are represented differently depending on their psychological distance from ourselves. Specifically, objects and events should be represented more abstractly the farther they are removed from direct experience through distance in the spatial, temporal, social, or hypotheticality domains. Bar-Anan et al. reported a key finding supporting this assumed association of the various distance dimensions and abstraction level. In their study, participants responded faster in an Implicit Association Task when temporally near and concrete concepts, as well as temporally far and abstract concepts, were mapped to the same rather than different response keys. In this study, we conceptually replicated this basic finding when employing temporal adverbs relating to present versus future time, and nouns referring to concrete versus abstract concepts (Experiment 1). Evidence for such an association, however, was largely absent (and significantly weaker than in Experiment 1) when temporal adverbs relating to the past were employed as instances of the large temporal distance category (Experiment 2). We propose that the uncertainty associated with the future, as opposed to the past, might play an important role in this temporal asymmetry by increasing psychological distance.

How do we represent the world surrounding us? Construal level theory (CLT; Trope & Liberman, 2003, 2010) suggests that objects or events are represented differently depending on their psychological distance from ourselves. Accordingly, something far from us in terms of temporal, spatial, or social distance, or merely hypothetical or unlikely, will be represented more abstractly, with less detail and in a rather prototypical, essential, and general fashion than something close to us. For example, the prospect of potentially starting a new job in a few years might bring along very different ideas and thoughts than the prospect of starting one’s first workday on a new job today. In the temporally distant case, one might be much more prone to think about general aspects such as career goals, intentions, and personal achievements associated with the new position (e.g., “will I be moving upwards a hierarchy level, which general management skills will I need, will the job be fulfilling”) and less about concrete and means-related aspects of starting the new job (e.g., “when will I have to be there, what will my office look like, on what will I work first”).

More generally speaking, CLT suggests that with a larger psychological distance, an object or event and the specific context in which it will take place will be uncertain. Consequently, mental representations of such distant objects or events have to rely on categorical knowledge and thus contain more general, abstract, and essential—and thus context-invariant—features: They will be formed at a “higher construal level” (Trope & Liberman, 2010). In contrast, objects or events in short psychological distances are typically anchored in a known context. Thus, they will be represented in much more detail, emphasising concrete and specific features that become relevant in this specific context—in terms of CLT, such representations are comprised at a “lower level of construal.”

Functionally, it has been suggested that a high level of construal serves to “mentally traverse psychological distances” (Trope & Liberman, 2010, p. 442) associated with temporally, spatially, or socially distant or uncertain events. This idea fits well with the widespread notion that more abstract mental representations (e.g., higher-order categories, for example, the category “dog” compared with a specific breed or even a specific exemplar of a dog) involve less detail and more generalizable attributes and thus allow for predictive processes (Gilead et al., 2020; Kelter & Kaup, 2019).

There is ample empirical evidence regarding this proposed association between psychological distance and construal level (Liberman et al., 2002; Nussbaum et al., 2006; Sagristano et al., 2002). For example, when participants were presented with a list of objects associated with a camping trip and asked to sort these objects into categories of their own choosing, they formed fewer and broader categories when they were instructed that the camping trip would take place in a year rather than on the next weekend (Liberman et al., 2002). While the temporal domain seems to be the most-studied distance dimension in the context of CLT (McCrea et al., 2008; Sánchez et al., 2021; Soderberg et al., 2015; Trope & Liberman, 2003), similar results have been reported in numerous studies involving manipulations of spatial (e.g., Fujita et al., 2006) or social (e.g., Liviatan et al., 2008) distance, or distance in terms of hypotheticality or probability (e.g., Wakslak & Trope, 2009). Thus, the distance-dependency of construal level seems to be a general and stable phenomenon (for a meta-analytic overview, see Soderberg et al., 2015; but also see Maier et al., 2022, for a recent reanalysis indicating large heterogeneity and the presence of publication bias in CLT effects reported in the literature).

A relation between distance and construal level was also demonstrated on the level of reaction times (RTs) in an extensive study by Bar-Anan et al. (2006), employing the Implicit Association Test (IAT, Greenwald et al., 1998). In the first experiment of this study, participants were to classify Hebrew words (for convenience, all following examples are translated to English) implying close (e.g., “tomorrow”) or far (e.g., “old age”) temporal distance and words related to either a low (e.g., “specific”) or high (e.g., “general”) level of construal. Then, these four response categories were mapped to two response keys, such that either one key was to be pressed for close and concrete words and the other one for distant and abstract words (in terms of CLT, this comprises a “matching” response mapping) or one key was to be pressed for close and abstract words, and the other for distant and concrete words (a “mismatching” mapping). The results were clear-cut: RTs to words in the matching conditions were considerably shorter than in the mismatching condition, indicating an associative relation between temporal distance and abstraction.

The generality of this finding was established in the remaining experiments of Bar-Anan et al.’s (2006) study, in which consecutively, four distance dimensions (spatial, temporal, social distance, and hypotheticality) were combined with two different manipulations of construal level. Specifically, adverbs relating to abstract (e.g., “general,” “universal”) versus concrete (e.g., “specific,” “detailed”) features were employed in half of the experiments. By contrast, nouns indicating subordinate exemplars (e.g., “hammer,” “poodle”) versus superordinate category labels (e.g., “furniture,” “animals”) were employed in the other half of the experiments. In all experiments, matching response mappings yielded shorter RTs than mismatching response mappings. Thus, the association between psychological distance and construal level seems to be a stable phenomenon, stretching to different conceptualisations of abstraction or construal level and various distance dimensions.

Moreover, as Bar-Anan et al. (2006) suggested, their results seem to reflect a relation between distance and construal level on a conceptual level. Specifically, the IAT task does not require an active, intentional combination or interpretation of the probed associations. That is, typical studies testing the predictions of CLT often require the formation of an explicit relation between the tested word categories. For example, participants may be explicitly asked to interpret a given concept (e.g., categorise camping gear) within a specific context (e.g., for a trip taking place either tomorrow or a year later)—hence, typically an integration of the probed concepts and a specific situated context is required in such tasks. In the IAT task described above, no such integration is needed—the IAT task probes for associations between categories without explicitly requiring the participants to deliberately combine or integrate the categories (hence the term “implicit association task,” see Greenwald et al., 1998, 2021). That is, the RT benefit of matching category-to-response mappings in the IAT task is assumed to emerge independently of an active combination of the employed target concepts. This also raises the question about the exact nature of the associations probed in the IAT task and whether these associations are “implicit” themselves—for example, in the sense of emerging automatically or being accessible to conscious report. As this question is not crucial for our present aim, however, we will briefly come back to this in the “General discussion” section (for extended discussions and alternative interpretations of the mechanisms underlying the IAT, see De Houwer, 2001; De Houwer et al., 2009a, 2009b; Rothermund, 2001; Rothermund & Wentura, 2004).

In this study using the IAT task, we aim to conceptually replicate and extend the basic association between abstraction and temporal distance as reported by Bar-Anan et al. (2006). In our study, we will emphasise two issues relating to the psychological distance dimension of time. First, we will employ slightly different operationalisations of the concepts of abstraction and temporal distance, to investigate the generalisability of the proposed association of time and abstraction in the sense of a conceptual replication (Hudson, 2023). Second, across two experiments, we will aim for a more fine-grained analysis of the concept of temporal distance.

Regarding our first aim, we will investigate whether the association between time and abstraction will also hold when employing purely temporal terms (e.g., “today”, “future”) for manipulating distance, and a manipulation of abstraction level on the instance level (Reed, 2016). Specifically, in Experiment 1 of Bar-Anan et al. (2006), participants had to distinguish between “things that will happen soon” (as “eat,” “tomorrow,” “drink,” “conversation”) and things that will happen in a long time (as “old age,” “retirement,” “2009,” “PhD”). While these concepts can be distinguished based on their temporal occurrence, they might also be judged as more or less concrete or abstract events (e.g., “eat” is associated with more modal and experiential qualities than “retirement”). This might have contributed to the observed association between temporal distance and abstraction level. A more straightforward relation of the word material to time was given in their Experiment 2 (near time: “a second,” “a minute,” “now,” “immediately,” “soon” vs. distant time: “a year,” “a decade,” “later,” “last year,” “long ago”). Notably, however, here temporal distance was implemented in terms of time periods (e.g., “a minute,” “a year”) as well as words referring to the near or far future (e.g., “soon,” “later”) and past (“long ago”). Thus, it remains somewhat unclear which of these instances of distance contributed to the observed association between time and abstraction. Therefore, we aim to conceptually replicate these experiments by manipulating temporal distance through temporal adverbs that clearly relate to the present, the future, or the past.

Furthermore, according to Reed (2016), abstractness is not a unitary concept but can be conceptualised at different levels: attributes, categories, and instances. For example, a representation will be more abstract if certain attributes of its referent are retained while other attributes (e.g., task-irrelevant ones) are discarded. In this sense, abstract representations would be more global, universal, or schematic and thus retain only essential properties of the referent, rather than concrete, detailed, or specific (e.g., as in a sketch of a chair compared with an image of this chair). Likewise, on the level of categories, a particular, concrete object (e.g., a chair) could also be represented more abstractly as a member of a higher-level category or class (i.e., as a piece of furniture) based on its similarity to other members of this class. Finally, on the level of instances, abstraction refers to the nature of the referent itself: Here, a concept can be regarded as abstract if it does not possess a material referent. For example, the concept “chair” would be more concrete than the intangible concept “effort.” Whereas Bar-Anan et al. (2006) manipulated abstractness on the level of attributes (e.g., using terms as “detailed” or “general”) or categories (e.g., using terms as “poodle” or “animals”), we will employ a manipulation based on the instance level postulated by Reed (2016). Specifically, we will present nouns that either have a material referent (e.g., “charcoal,” “window”) or not (e.g., “principle,” “belief”), as low- and high-abstraction-level stimuli, respectively. This will allow generalising the basic effects observed by Bar-Anan et al. to Reed’s complete taxonomy of abstraction.

The second aim of this study is related to several recent studies that investigated a particular feature of egocentric (or deictic) time. Specifically, in our subjective experience, time seems to unfold from the present in two directions: the future and the past. Interestingly, while “clock time” runs steadily and homogeneously, implying a symmetry of deictic time as it unfolds from the egocentric present (e.g., yesterday and tomorrow are equidistant from the present), psychologically, the past and the present go along with certain asymmetries in cognitive processing. For example, future actions are typically valued higher than past actions (Caruso et al., 2008). Thinking about the future is more personally involving and less constrained by situational context than thinking about the past (Pronin & Ross, 2006; Van Boven & Ashworth, 2007), and participants focus on different features of a task or situation when thinking about past and future performances (Ferrante et al., 2013). Interestingly, Kane et al. (2012) report that in mental time travel, imagined future events or actions are represented more prototypically and abstractly than imagined past events or actions. Presumably, this results from retrospection being more grounded in contextual knowledge and more constrained by factual memories than prospection.

While CLT explicitly assumes that increasing temporal distance, be it towards the past or the future, should yield more abstract cognitive representations (Trope & Liberman, 2003), even within this framework, such psychological asymmetries might be readily explained. As the future is typically unknown and thus more uncertain than the past, the future may not only be distant in terms of temporal distance but also regarding the dimension of hypotheticality. This implies that participants would construe objects or events in the future more abstractly than in the equidistant past (see also Kane et al., 2012). Therefore, in two experiments, we will examine whether the directionality of the temporal distance (present vs. future vs. present vs. past) affects the association between distance and construal level in the IAT task.

## Experiment 1

### Methods

In this experiment, we assessed the association between abstraction and future time in an IAT task. Abstraction level was manipulated by presenting nouns corresponding to abstract versus concrete concepts, and temporal distance was manipulated by presenting temporal adverbs corresponding to present or future time. According to CLT, one would expect that shorter RTs emerge when the categories “abstract” and “future” are mapped to one response key and the categories “concrete” and “present” are mapped to another response key (matching condition) than when the categories “abstract” and “present” are mapped to one key and “concrete” and “future” are mapped to the other response key (mismatching condition). This and the following experiments were ethically approved prior to data collection by the Ethics Committee for Psychological Research of University of Tübingen (Kaup_2021_1007_239/Kaup_2021_0623_230). The hypotheses, methods, and data analysis plan for this experiment were preregistered prior to data collection (Open Science Framework, https://doi.org/10.17605/OSF.IO/WH7RU). Deviations from preregistrations, if any, will be explicitly stated in the text. The full data sets and coding information for this and the following experiments can be downloaded in the associated OSF project (Bausenhart, 2023).

As outlined in the introduction, this experiment aims to conceptually replicate (cf. Hudson, 2023) Experiments 1A and 1B of Bar-Anan et al. (2006). That is, we will reinvestigate the association between time and abstraction by means of the IAT task while varying the exact operationalisation of the experimental variables and details of the procedure, to examine the generality of this basic phenomenon. To allow for a comparison of our and Bar-Anan et al.’s experiments, the most prominent differences between our experiments and the respective experiments of the original study are summarised in Table 1. In addition to the intended conceptual differences in the operationalisation of our variables, most of these variations (e.g., sample size, number of items and trials, analysis method) aimed at increasing the stability of our results and the power of our experimental design.

**Table 1.** Main deviations between Experiments 1A and 1B of Bar-Anan et al. (2006) and the experiments of this study.

	Bar-Anan	Present study
Temporal distance	Temporally near events versus temporally distant events (Exp. 1A) Adjectives and nouns relating to present and short timeframes versus future, past, and long timeframes (Exp. 1B)	Temporal adverbs relating to present versus future time points (Exp. 1) Temporal adverbs relating to present versus past time points (Exp. 2)
Abstraction level	Adjectives relating to abstract or concrete features (Exp. 1A) Exemplars versus categories (Exp. 1B)	Concrete and abstract instances (Exp. 1 & 2)
Stimulus language/participant first language	Hebrew	English
Number of items per category	*n* = 4 (Exp. 1) *n* = 5 (Exp. 2)	*n* = 6 (Exp. 1 & 2)
Sample size	*N* = 16 (Exp. 1A) *N* = 12 (Exp. 1B)	*N* = 96 (Exp. 1 & 2)
Number of trials per participant (experimental blocks only)	*n* = 64 (Exp. 1A) *n* = 80 (Exp. 1B)	*n* = 240 (Exp. 1 & 2)
Testing conditions	Laboratory setting	Online experiment
Main dependent variable and analysis	D (cf. Greenwald et al., 2003), t-tests	RT, linear mixed-effects model

Besides the differences mentioned in Table 1, some minor methodological differences should also be noted. For example, in contrast to the original study, we used different response keys (I vs. E in Bar-Anan; x vs. m in the present study). In addition and unlike in Bar-Anan’s original experiments (and customary in many IAT studies), we decided not to omit the repeated practice block of the nonvaried word type in the second half of the experiment, to keep the number of occurrences of each word constant for the matching and mismatching conditions. In addition, we balanced the category-to-response-key mappings completely: For part of our sample, the response mapping for abstraction level was kept constant and varied for temporal distance, and vice versa for the other part (see below for details). In contrast, in Bar-Anan’s original study, the response mapping for abstraction level was always kept constant, and varied for temporal distance only. Due to the online testing situation, we employed visual instead of auditory error feedback. Our stimuli were presented in white (instead of blue as in Bar-Anan et al.) on a black background, and while no explicit data rejection criteria were mentioned in Bar-Anan, we excluded participants with more than 25% of errors and trials with exceptionally long RTs (>4 s) and RTs outside of *M* *±* 3 *SD* range per participant and condition. Although this is undoubtedly an empirical issue, we deem these and potentially other more minor methodological differences between the two studies as relatively inconsequential for the general pattern of results.

#### Participants

The sample size was determined a priori through data simulation. Specifically, we simulated RT data for a linear mixed-effects model analysis, corresponding to the one described in the “Results” section of this experiment, by using the mixedpower package for R (Kumle et al., 2021). Parameters for this simulation (beta estimates, random effects variances, and residual standard deviation) were based on pretest data, and we aimed at a minimum effect of interest of 35 ms (RT mismatch – RT match) for the simulation. The power simulation yielded a minimal sample size of 88 participants to achieve a power of .86. For comparison, in a conventional analysis (paired-samples *t* test), a sample of 88 participants would be sufficient to detect a 35 ms difference with an associated *SD* of 107 ms (i.e., Cohen’s *d* = 0.33), with a power of .86. We then conservatively added ~ 10% to the sample size estimate, resulting in a required sample size of 96 participants.

We recruited participants through the online platform “Prolific” (www.prolific.co), with the following restrictions: minimum age of 18 years, English as first language, and a current location in Great Britain or the United States. The data from three participants were excluded because of a large proportion of erroneous responses (>25% of trials). The final sample consisted of 96 participants (58 females, 37 males, and one who did not specify their gender), with a mean age of 42.1 (*SD* = 13.4, range = 18–73) years. According to self-report, 84 were right-handed, 10 left-handed, and two were ambidextrous. Only complete data sets were used for analysis. All participants provided informed consent through a checkbox on an experiment information page before starting data collection.

#### Stimuli and apparatus

Overall 24 English words (see Table 2) were employed as stimuli, resulting in six words for each level of Word Type (Time vs. Abstraction) and Distance/Construal Level (small/low vs. large/high). These words were selected through the following procedure: Twelve time words (six present, six future) were selected with the restriction that only unambiguous English one-word expressions relating to present or future time were used. Then, we matched six abstract and six concrete English nouns to these time words. Specifically, we made a preselection of English nouns with at least three letters and which were rated with a mean concreteness of either > 4.5 or <1.5, and a *SD* of these ratings smaller than 0.8, from the corpus of concreteness-rated words provided by Brysbaert et al. (2014). From this preselection, we chose abstract and concrete nouns that best matched the 12 time words according to standardised word frequency indices such as SUBTLWF (word frequency per one million words, from a database of approximately 51 million words collected through television and film subtitles) and the ZIPF scale value (logarithmic word frequency expressed through a 7-point scale, cf. van Heuven et al., 2014), and the number of letters, as taken from the SUBTLEX-US corpus (Brysbaert & New, 2009). This matching was accomplished by using the “anticlust” package for R (Papenberg & Klau, 2021). The resulting word selection did not differ significantly between the four word types concerning ZIPF, SUBTLWF, number of letters, and number of syllables, as computed conservatively with uncorrected pairwise *t* tests (all *p*s > .05).

**Table 2.** Word stimuli employed in Experiment 1.

Word type	Distance / Construal level	Words
Time	small / low	instantly, currently, presently, immediately, today, now
	large / high	upcoming, afterwards, future, tomorrow, later, next
Abstraction	small / low	charcoal, wheelchair, window, daughter, door, letter
	large / high	sanctity, principles, goodness, possibility, concept, belief

The experiment was programmed in jsPsych (De Leeuw, 2015) and run online through the participants’ private desktops or laptops. Responses were collected through the “x” and “m” keys of their keyboards.

#### Procedure

In each trial of this experiment, a single word was presented in white on a black background at the screen centre until participants responded with a single key press of their left (key “x”) or right (key “m”) index finger.

At the beginning of the experiment (and once the key assignment changed), participants received written instruction prior to each practice block that they would see “time-related words that either describe the present or the future,” “words that either describe something concrete or something abstract,” or “both types of words intermixed.” They were instructed to press one key for time words relating to the “future” and the other key for time words relating to the “present,” and to press one key for “concrete” words and the other key for “abstract” words. In addition to these category labels, the instruction for the practice blocks also showed all word exemplars and their category assignment (e.g., *Present words are: today, instantly, currently, now, immediately, presently*), to inform the participants fully and to avoid initial confusion about the word-to-category assignment.

The specific assignment of words to keys was counterbalanced between participants and varied after half of the trials for one type of words (see below for details). In general, however, each participant performed one half of the experiment with a matching stimulus-response assignment (i.e., present/concrete matched to one key and future/abstract to the other key), and one half of the experiment with a mismatching assignment (i.e., present/abstract matched to one key and future/concrete to the other key). In case of a correct response, the next word was presented after an intertrial interval of 250 ms. In case of an erroneous response, the word *error* and a reminder of the specific stimulus-response key assignment of the current block were presented for 2,500 ms before the intertrial interval.

All participants performed eight blocks of trials, with a self-terminated break between blocks. Blocks 1 to 3 were designated to practice the stimulus-response mappings for time words in isolation (12 trials in random order, each time word presented once), for abstraction words in isolation (12 trials in random order, each abstraction word presented once), and for both word types randomly intermixed (24 trials in random order, each word presented once). In Block 4, all 24 words were presented in random order, and this was repeated 5 times (120 trials). Therefore, as in Bar-Anan et al.’s (2006) study, the same word type could repeat for multiple trials.^1^ Then, the stimulus-response mapping was changed for one word type, and the participants again performed three blocks of practice and one experimental block.

Participants were randomly assigned to one of eight different experimental versions for counterbalancing. First, these versions differed in the order of the Match conditions (i.e., matching stimulus-response assignment first vs. nonmatching stimulus-response assignment first). Second, we counterbalanced the word type for which stimulus-response assignment was varied after half of the experiment (i.e., assignment for time words varied vs. assignment for abstraction words varied). The varied word type was always practised in isolation in Blocks 1 and 5, and the nonvaried word type was practised in isolation in Blocks 2 and 6. Finally, the specific stimulus-response assignment of the nonvaried word type was also balanced over participants (i.e., “respond left” to present [concrete] words vs. “respond right” to present [concrete] words).

#### Analyses

As preregistered, we performed linear mixed-effects model analyses to investigate the influence of Match, Word Type, and their interaction as fixed effects on response accuracy and RT. These analyses were conducted with the lme4 package for R (Bates et al., 2015), using the glmer function with a logit link function for response accuracy, and the lmer function for RT. All predictors were sum-coded, and we analysed full models including random effects for participants (random intercept and slopes for Match and Word Type) and word (random intercept and slope for Match) first. In case of convergence issues, different optimizers were tested using the “all_fit” function from package afex (Singmann et al., 2016), and subsequently, the random effects structure would be simplified until convergence. The fixed effects of the resulting model were further evaluated through model comparison using the mixed function of the afex package. To complement the mixed-effects model analyses, conventional F1, F2, and Fmin’ (Clark, 1973, Equations 15 and 16) analyses of variance were conducted for the percentage of correct responses (PC) and RT.

#### Results

Practice trials did not enter data analyses. As specified above, three participants with overall less than 75% of correct responses in the experimental blocks were excluded from data analysis. On a single-trial basis, we excluded outliers based on a two-step procedure. First, we excluded all trials with RTs > 4 s (0.38% of trials), assuming that these long RTs must be due to lapses of attention (e.g., participants taking a break or being distracted during the task). Then, we excluded additional trials if RT in these trials was outside a *M* *±* 3 *SD* range (2.02% of trials), with *M* and *SD* computed separately from correct trials of each participant and experimental condition (each combination of Match × Word Type).

**Figure 1.**
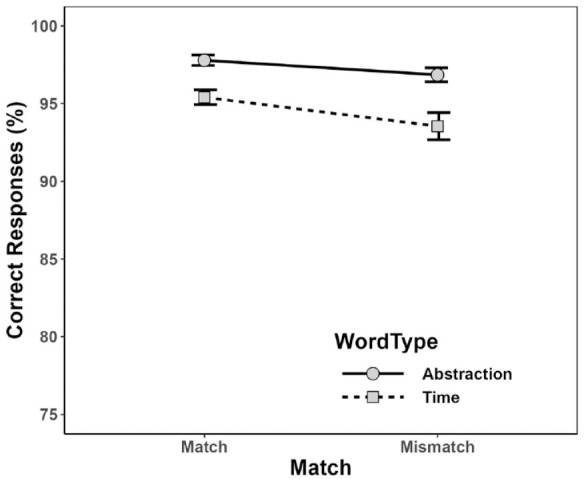
Mean percentage of correct responses (PC) for each combination of Word Type and Match in Experiment 1. Error bars depict ±1 *SE.*

**Figure 2.**
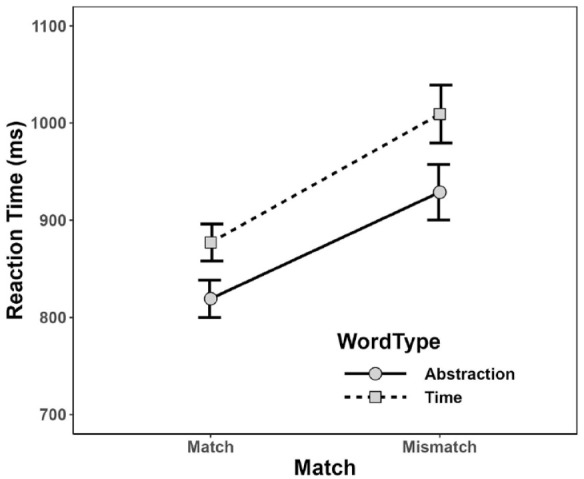
Mean reaction time for each combination of Word Type and Match in Experiment 1. Error bars depict ±1 *SE.*

Figures 1 and 2 depict mean PC and mean RT, calculated for the combination of Word Type and Match condition. As can be seen, participants tended to elicit more correct responses for abstraction words (*M* = 97.3%, *SD* = 2.93%) than for time words (*M* = 94.5%, *SD* = 5.86%), and in matching (*M* = 96.6%, *SD* = 2.93%) than in mismatching (*M* = 95.2%, *SD* = 5.91) conditions. In addition, mean RT was considerably shorter for abstraction words (*M* = 874 ms, *SD* = 220 ms) than time words (*M* = 943 ms, *SD* = 223 ms), and in matching (*M* = 848 ms, *SD* = 177 ms) than in mismatching (*M* = 969 ms, *SD* = 269 ms) conditions.

##### Mixed-effects model analyses

To check this result pattern statistically, we performed a linear mixed-effects model analysis in which we investigated the influence of Match, Word Type, and their interaction as fixed effects on response accuracy. The full mixed-effects binomial logistic regression model for accuracy as dependent variable (DV),

$$\begin{array}{l} \mathrm{DV}\sim 1+\mathrm{Match}*\mathrm{Word}\;\mathrm{Type}+\\ \;\;\;\;\;\;\;\;\;\;\;\;\;\;\left(1+\mathrm{Match}*\mathrm{Word}\;\mathrm{Type}|\mathrm{Participant}\;\right)\\ \;\;\;\;\;\;\;\;\;\;\;\;\;\;+\left(1+\mathrm{Match}|\mathrm{Word}\right) \end{array}$$

converged using the bobyqa optimizer. It yielded a significant effect of Word Type (β = 0.42, *SE* = 0.09, *z* = 4.73, *p* < .001), reflecting more correct responses for abstraction than for time words. Neither Match (β = 0.13, *SE* = 0.08, *z* = 1.59, *p* = .11) nor the interaction of the two predictors (β = 0.05, *SE* = 0.08, *z* = 0.70, *p* = .483) reached significance. Confirming these results, model comparisons with its nested models showed that only factor Word Type contributed significantly to improving the model fit (χ² = 18.47, *p* < .001, all other *p*s > .12).

An analogous linear mixed-effects analysis (Gaussian link function) for RT as a dependent variable also converged using the bobyqa optimizer. It also yielded a significant effect of Word Type, β = –34.01, *SE* = 12.81, *t*(44.39) = -2.66, *p* = .011, reflecting faster responses for abstraction than for time words. Most importantly, there was a significant effect of Match, β = –60.34, *SE* = 8.92, *t*(96.15) = –6.76, *p* < .001, with faster responses in the matching than in the mismatching condition. There was no interaction between the two predictors, β = 5.44, *SE* = 4.14, *t*(74.31) = 1.31, *p* = .193. Confirming these results, a hierarchical comparison of this model with its nested models showed that Word Type (χ² = 6.52, *p* = .011) and Match (χ² = 37.47, *p* < .001), but not their interaction (χ² = 1.71, *p* = .192), contributed to improving the model fit.

##### ANOVA analyses

The analyses above were complemented by conventional F1, F2, and Fmin’ analyses of variance (ANOVA) on PC and mean RT. The complete results of these analyses are listed in Table 3. As can be seen, the results of these analyses mirror the results of the linear mixed effects modelling quite closely, showing consistent main effects of Word Type for both PC and mean RT, as well as a clear main effect of Match for mean RT. In slight contrast to the linear mixed effects modelling, a main effect of Match was also observed for PC in these ANOVA analyses.

**Table 3.** Results of F1, F2, and Fmin’ analyses of variance for Experiment 1.

	F1				F2				Fmin’		
	*df*	*F*	*p*	$\eta_{p}^{2}$	*df*	*F*	*p*	$\eta_{p}^{2}$	*df*	*F*	*p*
*Percentage of correct responses*											
Word Type	1, 95	30.18	**<.001**	.24	1, 22	31.79	**<.001**	.59	1, 73.79	15.48	**<.001**
Match	1, 95	6.97	**.010**	.07	1, 22	9.59	**.005**	.30	1, 86.30	4.04	**.048**
Word Type × Match	1, 95	1.65	.202	.02	1, 22	1.03	.320	.04	1, 53.31	0.64	.427
*Mean reaction times*											
Word Type	1, 95	22.51	**<.001**	.19	1, 22	10.33	**.004**	.32	1, 44.64	7.08	**.011**
Match	1, 95	45.78	**<.001**	.33	1, 22	693.06	**<.001**	.97	1, 105.97	42.95	**<.001**
Word Type × Match	1, 95	1.91	.170	.02	1, 22	7.48	**.012**	.25	1, 116.82	1.52	.220

*Note:* Boldface indicates statistical significance.

#### Discussion

As expected, and replicating the pattern of results observed by Bar-Anan et al. (2006), responses were faster (and tended to be more correct) in matching than in mismatching conditions. Specifically, participants responded faster when present and concrete words were assigned to one response key and future and abstract words to the other response key, than when present and abstract words were assigned to one key and future and concrete words to the other key. This supports the assumption of an association between temporal distance and abstraction level central to CLT (Trope & Liberman, 2003, 2010), namely that small temporal distance is related to more concrete representations and large temporal distance is related to more abstract representations. Our study thus extends previous results to English stimulus material and to a manipulation of abstraction at the instance level (cf. Reed, 2016). Specifically, unlike Bar-Anan, we employed words for entities that can be described as more or less abstract. These instances may be distinguished on the grounds of having a material referent or not (e.g., “charcoal” vs. “sanctity”), and were clearly distinguishable based on abstraction ratings readily available in current literature (Brysbaert et al., 2014). Moreover, we manipulated temporal distance with purely temporal adverbs that could be categorised as either present or future time points (e.g., “now” vs. “next”). Interestingly, although most of these temporal adverbs only specify a rather small or unclear magnitude of the temporal difference between the categories “present” and “future” (e.g., one day in case of “today” vs. “tomorrow”), this was sufficient to elicit a substantial response time difference (around 121 ms) between matching and mismatching response key assignments. In contrast, Bar-Anan’s stimuli were at least in part allocated on a much larger time scale: for example, “a minute”, “eat” for close temporal distance and “a decade”, “retirement” for large temporal distance. Thus, in light of the relatively small temporal distance range employed in the present experiment, one might speculate that assigning a temporal word to the categories “present” or “future” is already sufficient to elicit an association between temporal distance and abstraction, despite the specific magnitude of this distance. This may suggest that the distinction between “close” and “far” temporal distance is categorical rather than ordinal or even linear. It would be interesting for future research to see how the magnitude of distance affects the relationship to abstraction level.

Another open issue, as outlined in the introduction, is that the present experiment employed only words relating to present versus future time points. Interestingly, these two categories do not only differ in terms of their temporal distance. In fact, according to the conceptualisation of psychological distance employed in CLT, the future is not only psychologically distant because it is temporally farther from the present, but also because it is unknown and thereby uncertain. This adds a second proposed dimension of psychological distance, hypotheticality, to our experimental manipulation. Consequently, the uncertainty typically associated with the future might have contributed to the association between time and abstraction observed in the present study. In Experiment 2, we manipulated temporal distance by employing temporal adverbs exclusively relating to present and past time points to investigate this issue further.

#### Experiment 2

##### Methods

We again assessed the association between abstraction and time in this experiment. The only deviation from Experiment 1 was that the temporally far condition was now implemented by presenting temporal adverbs relating to the past instead of the future. If temporal distance towards the past alone is sufficient to create psychological distance, one would again expect shorter RTs when the categories “abstract” and “past” are mapped to one response key and the categories “concrete” and “present” are mapped to another response key (matching condition) than when the categories “abstract” and “present” are mapped to one key and “concrete” and “past” are mapped to the other response key (mismatching condition). However, if the uncertainty associated with the future also plays a role in the results observed in Experiment 1, the association between abstraction and time should be less pronounced in Experiment 2 than in Experiment 1. Hypotheses, methods, and data analysis plan for this experiment were also preregistered prior to data collection (Open Science Framework, https://doi.org/10.17605/OSF.IO/672DB).

###### Participants

As in Experiment 1, complete data sets from 96 participants were collected through the online platform “Prolific,” with the same restrictions as in Experiment 1. The resulting sample comprised 72 women and 24 men, with a mean age of 38.4 (*SD* = 12.5, range = 18–72) years. Eighty-three were right-handed, 12 left-handed, and one ambidextrous. All participants provided informed consent through a checkbox on an experiment information page before starting data collection.

###### Stimuli, apparatus, procedure, design, and analyses

All stimuli and the procedure employed in Experiment 2 were identical to those of Experiment 1, with the only exception that large temporal distance was implemented by using six English temporal adverbs relating to the past instead of the future: “past,” “before,” “ago,” “yesterday,” “former,” and “previously.” All other words were the same as the ones employed in Experiment 1, and again it was ensured that the resulting word selection did not differ significantly between the four word types with respect to ZIPF, SUBTLWF, number of letters, and number of syllables, as computed conservatively with uncorrected pairwise *t* tests (all *p*s > .05).

##### Results

Practice trials did not enter data analyses. No participants were excluded according to the preregistered exclusion criteria. On a single-trial basis, we again excluded outliers based on a two-step procedure. First, we excluded all trials with RTs > 4 s (0.43% of trials), and second, we excluded additional trials (2.08%) if RT in these trials was outside a *M* *±* 3 *SD* range, with *M* and *SD* computed separately from correct RTs for each participant and experimental condition (each combination of Match × Word Type).

**Figure 3.**
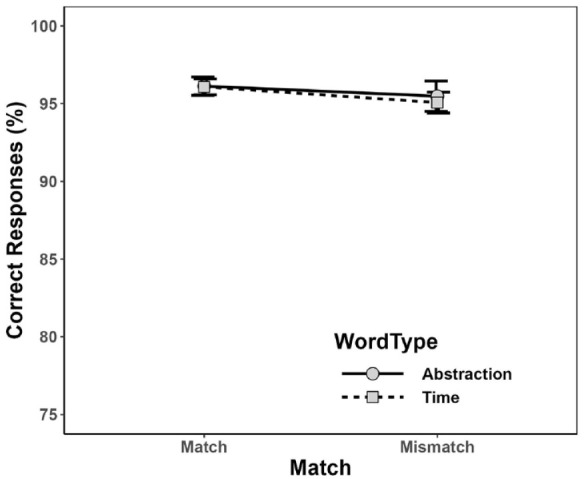
Mean percentage of correct responses (PC) for each combination of Word Type and Match in Experiment 2. Error bars depict ±1 *SE.*

**Figure 4.**
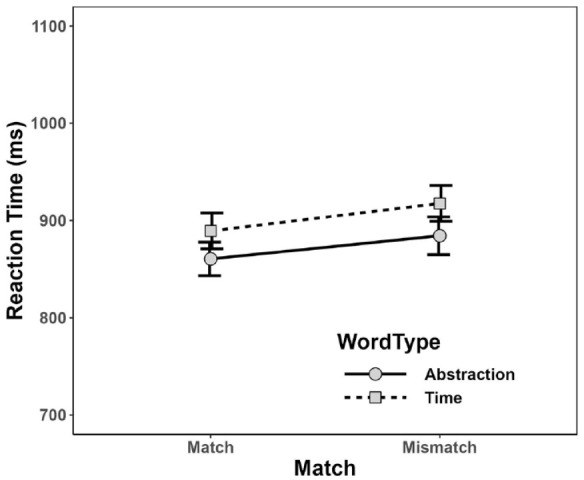
Mean reaction time for each combination of Word Type and Match in Experiment 2. Error bars depict ±1 *SE.*

As before, PC and mean RT were calculated for each combination of Word Type and Match condition, as depicted in Figures 3 and 4. As can be seen, participants elicited a similar PC for abstraction words (*M* = 95.8%, *SD* = 6.2%) and time words (*M* = 95.6%, *SD* = 4.4%), and also similar percentages in matching (*M* = 96.1%, *SD* = 4.7%) and in mismatching (*M* = 95.3%, *SD* = 7.5%) conditions. Numerically, mean RT was shorter for abstraction words (*M* = 872 ms, *SD* = 159 ms) than for time words (*M* = 903 ms, *SD* = 155 ms), and in matching (*M* = 875 ms, *SD* = 167 ms) than in mismatching (*M* = 901 ms, *SD* = 176 ms) conditions, but—in comparison with the results of Experiment 1—these differences seem considerably smaller.

###### Mixed-effects model analyses

To check the statistical significance of these effects, we again performed a linear mixed-effects model analysis in which we investigated the influence of Match, Word Type, and their interaction as fixed effects on response accuracy. The full mixed-effects binomial logistic regression model for accuracy as dependent variable (DV),

$$\begin{array}{l} \mathrm{DV\sim}1+\mathrm{Match}*\mathrm{Word}\;\mathrm{Type}+\\ \;\;\;\;\;\;\;\;\;\;\;\left(1+\mathrm{Match}*\mathrm{Word}\;\mathrm{Type}|\mathrm{Participant}\;\right)\\ \;\;\;\;\;\;\;\;\;\;\;+\left(1+\mathrm{Match}|\mathrm{Word}\right) \end{array}$$

converged using the bobyqa optimizer. It yielded a significant fixed effect of Word Type (β = 0.26, *SE* = 0.10, *z* = 2.69, *p* = .007), reflecting slightly more correct responses for abstraction than for time words. Neither Match (β = –0.04, *SE* = 0.09, *z* = -0.46, *p* = .644) nor the interaction of the two predictors (β = –0.12, *SE* = 0.08, *z* = -1.61, *p* = .108) reached significance. Confirming these results, model comparisons with its nested models showed that factor Word Type indeed contributed significantly to improving the model fit (χ² = 6.73, *p* = .009, all other *p*s > .12).

An analogous linear mixed-effects analysis (Gaussian link function) for RT as a dependent variable also converged using the bobyqa optimizer. None of the fixed effects reached significance, Word Type: β = –14.91, *SE* = 12.55, *t*(27.73) = –1.19, *p* = .245; Match: β = –12.73, *SE* = 8.20, *t*(96.36) = –1.55, *p* = .124, Word Type × Match: β = 1.41, *SE* = 3.92, *t*(68.42) = 0.36, *p* = .720. Confirming these results, model comparison with its nested models showed that none of the fixed effects contributed to improving the model fit (a *p*s > .12).

###### ANOVA analyses

The analyses above were again complemented by conventional F1, F2, and Fmin’ analyses of variance on PC and mean RT (Table 4). As can be seen, these results also do not provide evidence for consistent effects of Match or Word Type, as Word Type only affected RT in the F1 analysis (*p* < .001), and Match only affected RT in the F2 analysis (*p* < .001), but neither effect was evident in the decisive, combined Fmin’ analysis.

**Table 4.** Results of F1, F2, and Fmin’ analyses of variance for Experiment 2.

	F1				F2				Fmin’		
	*df*	*F*	*p*	$\eta_{P}^{2}$	*df*	*F*	*p*	$\eta_{P}^{2}$	*df*	*F*	*p*
*Percentage of correct responses*											
Word Type	1, 95	0.26	.609	< .01	1, 22	0.12	.731	.01	1, 44.72	0.08	.779
Match	1, 95	1.09	.298	.01	1, 22	2.79	.109	.11	1, 110.62	0.79	.376
Word Type × Match	1, 95	0.19	.666	< .01	1, 22	0.18	.679	.01	1, 68.60	0.09	.765
*Mean reaction times*											
Word Type	1, 95	14.06	**<** **.001**	.13	1, 22	1.61	.218	.07	1, 27.23	1.44	.240
Match	1, 95	2.53	.115	.03	1, 22	26.00	**<** **.001**	.54	1, 109.89	2.30	.132
Word Type × Match	1, 95	0.09	.771	< .01	1, 22	1.09	.308	.05	1, 107.64	0.08	.778

*Note:* Boldface indicates statistical significance.

###### Additional analyses of Experiments 1 and 2 (not preregistered).^2^

**Figure 5.**
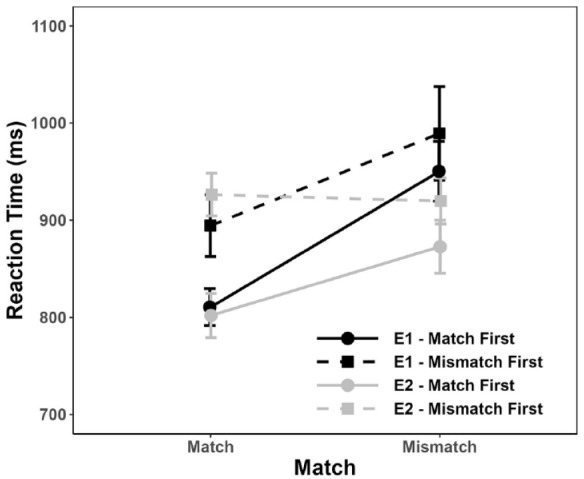
Mean reaction time for each combination of Match and Match order in Experiments 1 and 2. Error bars depict ±1 *SE.*

Experiments 1 and 2 suggest that the implicit association between Time and Abstraction is much stronger when “distant” time is implemented via future rather than past words. This, in turn, would be consistent with the notion that psychological distance implied by future words might be larger due to the additional uncertainty associated with future time. However, there are two caveats to this notion: First, it should be statistically investigated whether the Match effect observed in Experiment 1 is indeed significantly larger than the one observed in Experiment 2 (Gelman & Stern, 2006). Second, as outlined above, each participant was randomly assigned to one of eight experimental versions, through which the order of experimental conditions (e.g., matching condition presented in the first vs. matching condition presented in the second half) was varied. Unfortunately, this random assignment yielded an imbalanced number of participants per experimental version. For example, in Experiment 1, the matching stimulus-response assignment was received by 54 participants in the first half of the experiment, and by 42 participants in the second half. In Experiment 2, the matching assignment was received by 40 participants in the first half of the experiment and by 56 participants in the second half. As can be seen in Figure 5, this order affected the magnitude of the Match effect. Specifically, a larger Match effect was observed when participants started with the matching stimulus-response assignment in the first half and then relearned the mismatching stimulus-response for the second half of the experiment (solid lines), rather than vice versa (dashed lines, see also Greenwald et al., 1998, for a similar order effect).

It is thus conceivable that differences in the magnitude of the Match effect between Experiments 1 and 2 could be partially due to the more considerable difficulty of learning a mismatching response assignment after a matching one than vice versa. This could have increased the magnitude of the Match effect in Experiment 1, where more participants started with the matching assignment, and decreased the magnitude of the Match effect in Experiment 2, where more participants started with the mismatching assignment. To further investigate this issue, we conducted an additional analysis to complement the results of the planned analyses described above. Specifically, we compared the effect of Match on RT across experiments while controlling for the order of Match conditions (match first vs. mismatch first) as an additional fixed effect.

Specifically, we again performed a linear mixed-effects model analysis in which we investigated the influence of Match (match vs. mismatch), Experiment (E1: future vs. E2: past), and Match Order (match first vs. mismatch first), and their interactions as fixed effects on RT as DV. The following mixed-effects linear regression model, using a Gaussian link function,

$$\begin{array}{l} \mathrm{RT\sim}\;1+\mathrm{Match}*\mathrm{Match}\;\mathrm{Order}*\mathrm{Experiment}\\ \;\;\;\;\;\;\;\;\;\;+\left(1+\mathrm{Match}|\mathrm{Participant}\;\right)+\left(1+\mathrm{Match}|\mathrm{Word}\right) \end{array}$$

converged using the bobyqa optimizer (note that we did not include Match Order in the random effects structure due to convergence issues). It yielded a significant fixed effect of Match, β = –37.42, *SE* = 6.00, *t*(191.68) = –6.24, *p* < .001, reflecting faster responses in the matching condition than in the mismatching condition. Match Order also affected RT significantly, β = –36.59, *SE* = 12.99, *t*(191.72) = –2.82, *p* = .005, reflecting overall faster responses when participants started with the matching rather than the mismatching condition.

As outlined above, Match condition also interacted with Match Order, with a larger effect of Match when participants received the matching response assignment in the first half of the experiment rather than in the second half, β = –15.33, *SE* = 5.92, *t*(191.57) = -2.59, *p* = .010. While there was no fixed effect of Experiment, β = 9.65, *SE* = 13.01, *t*(193.33) = 0.74, *p* = .46, this predictor interacted with Match, β = –21.22, *SE* = 5.93, *t*(192.61) = -3.578, *p* < .001. Specifically, the Match effect was significantly less pronounced in Experiment 2 than in Experiment 1, thus corroborating the tentative conclusion based on the analyses reported above. None of the other interactions in the model were significant (*p*s > .50). To summarise, this analysis first suggests that the order of the matching conditions contributes to the magnitude of the Match effect. Second, however, and independent of this order effect, the effect of Match observed in Experiment 1 is larger than the one observed in Experiment 2.^3^

Finally, a similar mixed-effects model analysis conducted on the RT data from Experiment 2 alone, with model

$$\begin{array}{l} \mathrm{RT\sim}\;1+\mathrm{Match}*\mathrm{Matchorder}+\left(1+\mathrm{Match}\;|\;\mathrm{Participant}\right)\\ \;\;\;\;\;\;\;\;\;\;\;+\left(1\;+\;\mathrm{Match}|\mathrm{Word}\right), \end{array}$$

(optimizer bobyqa), showed significant effects of Match Order, β = –42.76, *SE* = 15.07, *t*(95.72) = -2.84, *p* = .006, and an interaction of Match Order and Match, β = –19.17, *SE* = 7.96, *t*(95.81) = -2.41, *p* = .018, but only a marginal effect of Match, β = –15.81, *SE* = 8.03, *t*(96.02) = -1.97, *p* = .052. Thus, when controlling for Match Order in Experiment 2, there is again no strong evidence for a Match effect.

##### Discussion

In Experiment 2, we did not observe clear evidence for an association between time and abstraction level. Specifically, although participants responded around 26 ms faster in the matching than the mismatching condition, this factor did not significantly contribute to the prediction of RT in the pre-planned linear mixed-effects analysis. Moreover, in the ANOVA analysis, the Match effect only reached significance in the “by item” (F2) analysis on RT, but not in the “by participant” (F1) or the decisive combined (Fmin’) analysis. These results suggest that “distant time,” when operationalised by temporal adverbs relating to past time, is not (or at least not strongly) associated with a high abstraction level. This contrasts with the results of Experiment 1, in which distant time was operationalised in terms of temporal adverbs relating to the future, and in which a clear effect of Match was observed. The different results between experiments were corroborated in additionally conducted analyses, which confirmed a larger influence of Match on RT in Experiment 1 than in Experiment 2 while controlling for Match Order (i.e., the order in which participants performed the different matching conditions).

#### General discussion

This study aimed to conceptually replicate previous evidence on the association between abstraction and time, as suggested by CLT (Gilead et al., 2020; Trope & Liberman, 2003, 2010). In Experiment 1, we presented adjectives relating to the present time and the future and nouns relating to concrete or abstract concepts. The four word categories were mapped to two response keys, such that either present time and concrete objects afforded a response with one key and future time and abstract objects afforded a response with another key (“matching” assignment), or such that present time and abstract objects afforded a response with one key and future time and concrete objects afforded a response with another key (“mismatching” assignment). Responses in the matching condition were reliably faster than in the mismatching condition, indicating an association between temporal distance and abstraction level as suggested by CLT (i.e., future associated with more abstract, and present associated with more concrete representations). This result is well in line with the findings of Bar-Anan et al. (2006) and extends them to a situation where temporal distance is manipulated via adverbs relating to either the present or the future, and where abstraction level is manipulated via nouns relating to either concrete or abstract concepts (the instance level according to Reed, 2016).

In Experiment 2, we investigated whether this association also holds for temporal distance as implied by adverbs relating to the past. Not only did the effect of Match fail to reach significance in our main analysis, but it was also significantly reduced compared with the one observed in Experiment 1. Thus, this indicates that the association between high abstraction level and future time is not mirrored by an equivalent association between high abstraction level and past time. A reason for this asymmetry might be that while the two experiments were set up for maximum comparability, the employed words for the temporally distant category necessarily differed between the two experiments. However, the overall low error rates suggest that the stimuli in both experiments could be classified sufficiently and comparably well as past/future or present words. Yet, it remains possible that the future words of Experiment 1 somehow implied a more precise temporal direction or a more considerable temporal distance than the past words of Experiment 2, thus allowing for a stronger association between time and abstraction in the former case. As most of the employed temporal adverbs (except for “tomorrow” and “yesterday”) did not imply absolute temporal distances from the present time, the resulting psychological distances evoked in participants’ perceptions are difficult to compare. Interestingly, however, it has been shown that participants tend to perceive future events as temporally closer than objectively equidistant past events (Caruso et al., 2013). Notably, such a tendency should have even counteracted the observed asymmetry of future and past in the present experiments.

In any case, compared with the relatively large temporal distances implied in Bar-Anan et al.’s original study (e.g., “a decade”, “retirement”, for future words), it seems striking that even relatively small (“today” / “tomorrow”) or merely categorical (“now” / “later”) temporal distances were sufficient to evoke a pronounced Match effect in Experiment 1. Presumably, it is rather the categorisation as “present” or “future” that is reflected in the observed association between temporal distance and abstraction level than the specific temporal distance of the exemplar stimuli (see also De Houwer, 2001; Mitchell et al., 2003). Therefore, the present results seem to contribute to a claim raised initially by Bar-Anan et al. (2006) that perceived distance is not (or not only) processed on an absolute scale, but is “determined by the context” (p. 619). For example, “tomorrow” may be either temporally close or distant, depending on whether it is compared with “today” or with “next year.” It remains up to future research to investigate the timescale and malleability of perceived distance in more detail. For example, an association between past time and high abstraction level similar to that of future time and high abstraction level may become more evident when employing larger temporal distances (just as one’s memory of their childhood may be more vague than one’s memories of last summer).^4^ Likewise, it seems plausible that, for instance, increasing the absolute temporal distance of future items even more might lead to a further increase in the observed association effect.

As we have outlined in the introduction, an essential factor presumably involved in the asymmetry of past and future’s association with abstraction level is embedded in time’s flow itself: The past is usually known, while the future remains open—it is associated with uncertainty, and thus, dealing with future events will require stronger abstraction from concrete context and more predictive processing (see also Kyung et al., 2014, for a potential moderating role of knowledge/uncertainty in the relation of temporal distance and abstraction). Therefore, it is likely that processing distant events would rely more on relatively stable and generalisable abstract features than on detailed, concrete, and modal representations (Gilead et al., 2020; Henderson et al., 2006; Nussbaum et al., 2006). From this point of view, it seems unsurprising that future time points are associated with abstract representations more strongly than equidistant time points in the past. In terms of CLT, this is consistent with the idea that psychological distance may emerge through a variety of distance dimensions, and that the future is not only distant along the deictic time axis but also along the dimension of certainty/hypotheticality (Kyung et al., 2014; Wakslak & Trope, 2009). Moreover, the observed asymmetry of the association between abstraction level and past versus future temporal distance also fits well with the results of several studies providing evidence for temporal asymmetries in other domains of cognitive processing, as value discounting (Caruso et al., 2008), personal involvement (Pronin & Ross, 2006; Van Boven & Ashworth, 2007), or mental time travel (Kane et al., 2012).

This study employed the IAT paradigm to investigate the association between abstraction and temporal distance. We would like to emphasise that, despite its popularity and widespread use in empirical research (e.g., Greenwald et al., 2003; Mitchell et al., 2003), the notion that the IAT reflects implicit cognitive associations between categories has been subject to some criticism (e.g., De Houwer, 2001; De Houwer et al., 2009a, 2009b; Meissner et al., 2019; Rothermund, 2001; Rothermund & Wentura, 2004). Specifically, it is under debate whether or to what extent the IAT represents an *implicit* measure (i.e., reflecting the outcomes of automatic, unintentional, and/or unconscious processes), and what the exact nature of the probed *associations* is. For example, it has been argued that rather than evaluating each item according to its designated category, participants might reduce the complexity of the task through recoding processes (e.g., a temporal adverb as “now” might be categorised as being more concrete than “soon”—thereby reducing the number of to be remembered category-to-response mappings in matching blocks). Similar recoding might also arise through category salience (e.g., the present might be more salient than the future, and concrete objects might be more salient than abstract objects or vice versa). Especially short RTs might then be observed when both salient categories are mapped to the same key. To our minds, this alternative interpretation is more problematic for the original use of the IAT—where researchers typically aimed to assess implicit attitudes towards or stereotypes associated with a target dimension (e.g., colour or gender) by probing its association to an evaluative attribute dimension (e.g., good vs. bad, weak vs. strong). Even if such recoding processes contributed to the results of the present study, its results would still indicate that present and concreteness or future and abstractness share a commonality that enables recoding in a CLT-congruent way, whereas this does not (or to a much lesser extent) apply to the combination of past and abstractness. However, such an interpretation does not entail that a change within one dimension (e.g., distance) would necessarily affect the representation of information along the other dimension (e.g., abstractness) as CLT implies. A somewhat related criticism is that the implicit associations, as supposedly assessed through the IAT, lack external validity because they are often not strongly predictive of behavioural criteria (Greenwald et al., 2009; Oswald et al., 2013).

Future studies might address these points explicitly. On one hand, one might employ advanced experimental setups such as the recoding-free IAT (Rothermund et al., 2009), which prevents recoding by randomly switching between matching and mismatching assignments. On the other hand, the lack of predictive value might be addressed by testing and comparing the asymmetric relation between abstraction level and temporal distance towards the future and past more directly (rather than focussing on supposedly implicit associations). Specifically, and as outlined in the introduction, if the temporal distance of a described event is manipulated experimentally, participants should form more or less abstract representations of the event and its features (and vice versa). According to the results of the present study, one would expect such an effect to be more pronounced for temporal distances extending to the future rather than to the equidistant past. Intriguingly, however, the majority of published studies reporting such influences has employed future-directed manipulations (typically comparing near-future with far-future events, for an overview, see Soderberg et al., 2015), whereas the analogous investigation of past-directed distance effects has received much less attention—a research gap that, based on the present results, should deserve more attention in future research.

In sum, the present results confirm Bar-Anan et al.’s (2006) original results that responses are faster when temporally near and concrete concepts, and temporally far and abstract concepts, are mapped to the same rather than different response keys. This suggests that temporal distance and construal level are cognitively related, as suggested by CLT. However, in this study, this relation only emerged when the large temporal distance was operationalised through temporal adverbs referring to the future, but was largely absent when temporal adverbs relating to the past were employed as instances of the large temporal distance category. We propose that the uncertainty associated with the future, as opposed to the past, might play a crucial role in this temporal asymmetry by increasing psychological distance.

## Supplemental Material

sj-pdf-1-qjp-10.1177_17470218231217732 – Supplemental material for Association between abstraction level and time: Are future and past more abstract than the present? Supplemental material, sj-pdf-1-qjp-10.1177_17470218231217732 for Association between abstraction level and time: Are future and past more abstract than the present? by Karin M Bausenhart, Rolf Ulrich and Barbara Kaup in Quarterly Journal of Experimental Psychology

## Notes

1. This deviates from Greenwald et al.’s (2022, see also Greenwald et al., 1998) best-practice suggestion to alternate between word types (i.e., between the two stimulus categories) rather than presenting them at random. According to these authors, alternating between the stimulus categories typically produces stronger Implicit Association Test (IAT) effects than the randomisation procedure. Accordingly, the present experiments might, as the ones in Bar-Anan et al.’s (2006) original study, result in somewhat conservative estimates of the association strength of time and abstraction.

2. As pointed out during the reviewing process, typical IAT data are often analysed in terms of the measure D (Bar-Anan et al., 2006; Greenwald et al., 1998, 2003), which divides the difference between matching and mismatching conditions by their common standard deviation, and which thus bears similarity with the effect size measure Cohen’s *d*. To allow for a more direct comparison of our results with the outcome of other studies, we conducted an additional exploratory analysis in which we followed the “improved scoring algorithm” outlined by Greenwald et al. (2003, see Table 4). For Step 7 of this algorithm, we used the block mean + 2 * *SD* as a replacement for error latencies. The results of this analysis correspond mostly well with the results of our main analysis. The difference scores were calculated such that positive values of D indicate an association of concrete and near time and abstract and far time (i.e., a “matching” combination in the sense of CLT). Specifically, in Experiment 1, average D amounted to 0.37 (*SD* = 0.49), and differed significantly from 0, *t*(97) = 7.50, *p* < .001. Slightly deviating from the results of our main analysis, the D value in E2 also differed significantly from 0, *t*(95) = 2.73, *p* = .007, but, most importantly, seemed numerically greatly reduced (*M* = 0.152, *SD* = 0.54) compared with Experiment 1. This was confirmed by a significant independent-samples *t* test comparing D across Experiments 1 and 2, *t*(192) = 2.95, *p* = .004. For comparison, in Bar-Anan et al.’s (2006) original study, D amounted to 0.54 (*SD* = 0.47) in Experiment 1A and to D = 0.37 (*SD* = 0.31) in Experiment 1B, the latter being virtually identical to the results of our Experiment 1.

3. Alternatively, one might address the issue of unequal participant numbers per experimental version by choosing from the complete sample a subset of participants for each of the eight experimental versions in such a way that data are completely balanced for version number. For control purposes, we conducted a mixed-effects model analyses for RT on such a balanced data set (created by picking the first five participants for each Version and Experiment, overall *N* = 80). Despite the reduced sample size, the most important results of this analysis resemble those derived on the basis of the complete sample of participants. Specifically, Matching (*p* < .001), Matching Order (*p* = .003), and the interaction of Matching × Experiment (*p* = .008) significantly predicted RT, again indicating faster reactions for matching rather than mismatching conditions, for matching order first rather than mismatching order first, and a stronger matching effect in Experiment 1 than in Experiment 2. The complete results of this analysis are available as supplementary material.

4. We thank Yoav Bar-Anan for pointing this out.
