# Supplementary material for: Association between abstraction level and time: Are future and past more abstract than the present?
Source: Q J Exp Psychol (Hove). 2023 Dec 27;77(8):1595–609. doi: 10.1177/17470218231217732 (PMC11295401; doi:10.1177/17470218231217732)
Supplement: sj-pdf-1-qjp-10.1177_17470218231217732 – Supplemental material for Association between abstraction level and time: Are future and past more abstract than the present? [file sj-pdf-1-qjp-10.1177_17470218231217732.pdf]

<u>Supplementary Material for</u>

Association between Abstraction Level and Time:

Are Future and Past more Abstract than the Present?

Karin M. Bausenhart, Rolf Ulrich, & Barbara Kaup

University of Tuebingen

Results of alternative analysis balancing participant numbers by experimental version for both experiments.

For this analysis, we choose from the complete sample a subset of five participants for each of the eight experimental versions, in order to achieve a sample completely balanced for version number. The results of a mixed-effects model,

RT ~ 1 + Match * Match Order * Experiment + (1 + Match | Participant) + (1 + Match | Word),

(Gaussian link function, optimizer bobyqa), yielded the following fixed effects:

	β	*SE*	df	*t*	*p*
(Intercept)	897.75	21.99	106.61	40.83	< .001
Match (matching condition)	-43.63	8.67	82.17	-5.03	< .001
Match Order (matching first)	-59.05	19.21	79.91	-3.07	.003
Experiment (E1)	37.52	19.25	80.55	1.95	.055
Match * Match Order	-0.41	8.57	79.96	-0.05	.962
Match * Experiment	-23.22	8.58	80.29	-2.71	.008
Match Order * Experiment	-19.65	19.21	79.91	-1.02	.309
Match * Match Order * Experiment	6.29	8.57	79.96	0.73	.465
